# Kindness Media Rapidly Inspires Viewers and Increases Happiness, Calm, Gratitude, and Generosity in a Healthcare Setting

**DOI:** 10.3389/fpsyg.2020.591942

**Published:** 2021-01-20

**Authors:** David A. Fryburg, Steven D. Ureles, Jessica G. Myrick, Francesca Dillman Carpentier, Mary Beth Oliver

**Affiliations:** ^1^ Envision Kindness, East Lyme, CT, United States; ^2^ Children’s Dental Associates of New London County, East Lyme, CT, United States; ^3^ School of Dental Medicine, Harvard University, Boston, MA, United States; ^4^ Bellisario College of Communications, Pennsylvania State University, University Park, PA, United States; ^5^ Hussman School of Journalism and Media, University of North Carolina, Chapel Hill, Chapel Hill, NC, United States

**Keywords:** happiness, inspiration, media, elevation, caring, prosocial, compassion, kindness

## Abstract

**Background and Objectives**: Stress is a ubiquitous aspect of modern life that affects both mental and physical health. Clinical care settings can be particularly stressful for both patients and providers. Kindness and compassion are buffers for the negative effects of stress, likely through strengthening positive interpersonal connection. In previous laboratory-based studies, simply watching kindness media uplifts (elevates) viewers, increases altruism, and promotes connection to others. The objective of the present study is to examine whether kindness media can affect viewers in a real-world, pediatric healthcare setting.

**Methods**: Parents and staff in a pediatric dental clinic were studied. Study days were randomized for viewers to watch either original kindness media or the standard televised children’s programming that the clinic shows. Participants scored self-rated pre-media emotions in a survey, watched either media type for 8 min, and then completed the survey. All participants were informed that they would receive a gift card for their participation. After completion of the survey, participants were asked if they wanted to keep the card or donate it to a family in need.

**Results:** Fifty (50) participants completed the study; 28 were parents and 22 were staff. In comparison to viewers of children’s programming, participants who watched kindness media had significant increases in feeling happy, calmer, more grateful, and less irritated (*p* < 0.05), with trends observed in feeling more optimistic and less anxious. Kindness media caused marked increases in viewers’ reports of feeling inspired, moved, or touched (*p* < 0.001). No change was observed in self-reported compassion, although baseline levels were self-rated as very high. People who watched kindness media were also more generous, with 85% donating their honoraria compared to 54% of Standard viewers (*p* = 0.03).

**Conclusions**: Kindness media can increase positive emotions and promote generosity in a healthcare setting.

## Introduction

Stress, particularly psychological stress, is a prominent feature of modern life. Stress is especially high for Americans – the United States is ranked among the top 10 most stressed countries in the world (Gallup, 2019). Stressors such as personal and familial health, safety, and finances are significant concerns documented annually by the American Psychological Association (APA, 2019). Other major stressors include discrimination, loneliness, and the workplace.

Many of these stressors are intertwined and exist in combination with others. Before COVID-19, the majority of people in the United States (and elsewhere) have been subject to a lot of stress. COVID-19 has only increased the stress that people are experiencing (Park et al., 2020).

The stress or allostatic load (McEwen and Gianaros, 2011) that people shoulder is important both for quality of life and for mental and physical health. Stress is a major cause of, or contributor to, disease. From a mental health perspective, stress can cause or exacerbate anxiety, depression, sleep disturbances, and cognitive impairment. From a physical perspective, stress is linked to heart disease, stroke, asthma, hypertension, and diabetes and obesity, among others (Schneiderman et al., 2005; Cohen et al., 2007; Liu et al., 2017).

Healthcare providers are suffering from even greater stress loads, as evidenced by burnout, depression, and suicide rates approximately twice that of the general public. There are multiple reasons for this problem, which many organizations, including the US National Academy of Medicine, are trying to address (National_Academy_of_Medicine_Action_Collaborative_on_Clinician_Well-Being, n.d.).

Positive social connection has been shown to help buffer stress, likely in multifactorial ways (Cohen and Wills, 1985; Kikusui et al., 2006; Hennessy et al., 2009; Thoits, 2011). Positive interpersonal connection is generated by prosocial behavior (e.g., volunteerism, donations, and social support; Inagaki and Eisenberger, 2012; Keltner et al., 2014; Son and Padilla-Walker, 2020) or what otherwise could be called, kindness, caring, compassion, or generosity, among several related terms. Engaging in prosocial behavior also induces happiness which, in turn, can reinforce continued prosocial behavior (Aknin et al., 2012; Layous et al., 2017; Curry et al., 2018).

Part of the effect of prosocial behavior is to induce elevation, an “other-praising moral emotion” (Haidt, 2003) that is elicited when witnessing others engage in virtuous acts such as generosity, kindness, love, or selflessness. The uplifted feeling of elevation can manifest physical sensations, including tearfulness or a warm feeling in the chest (Haidt, 2003; Algoe and Haidt, 2009).

Kindness media, or media portrayals of people helping or supporting each other, has been shown previously to elevate viewers and promote interconnectedness in laboratory settings (Algoe and Haidt, 2009; Janicke and Oliver, 2017; Oliver et al., 2015, 2018). In turn, viewing kindness media can promote altruism (Schnall et al., 2010), supporting the concept that behavior as well as emotions can be affected by this type of media. Finally, kindness media has also been shown to promote greater acceptance and connection to people of other races, suggesting that the increased sense of connection can help transcend racial differences (Freeman et al., 2009; Janicke and Oliver, 2017; Krämer et al., 2017).

The gold standard of healthcare is a professional interaction that is a blended art of kindness and compassion with applied science. As such, we wanted to test whether kindness media could uplift and inspire both patients and providers in healthcare. Increasing positive emotions and expression of caring in this setting could affect both staff and patients, help with patient engagement, as well as affect the patient–provider encounter.

The aim of this first study was to test the acute impact of viewing kindness media on emotional responses and generosity in a real-world healthcare setting.

## Materials and Methods

This was a randomized, baseline- and comparator-controlled field study of the effects of kindness media on emotions and behavior in a pediatric clinical care setting. Both parents of the patients and staff of the clinic (Children’s Dental Associates of New London County, CT) who were 18 years of age or older were invited to participate. After parents registered their children and completed any necessary clinic paperwork, in consecutive order they were each provided a description of the study and interest to participate was solicited by a study team member.

The staff were separately provided a description of the study. Parents and the staff were studied on different days. Participation took place on the same day individuals provided their consent.

The study was performed in the waiting room. Study days were randomized to either the “Standard” (or usual) children’s commercial television programming selected by the clinic or kindness media. All viewing occurred on the same television in the waiting room. Kindness media was streamed onto the waiting room television using digital signage software (Playsignage, Inc).

The study was reviewed and approved by an independent investigational review board (IntegReview, Austin, TX).

### Kindness Media

The kindness media (called EnSpire™) is the product of Envision Kindness, a not-for-profit organization that collects, creates, and shares images and stories of kindness. The objective of Envision Kindness is to inspire and uplift viewers, reduce stress, and promote kindness, compassion, joy, and love.

The kindness media contains multiple visual and story-telling components that depict different acts or aspects of kindness incorporated into a video format. At the core are culturally and geographically diverse, still images of kindness that have been shared with Envision Kindness. Beyond technical quality, images are selected for these videos using three criteria. First, do they depict a positive act of kindness, connection, or caring? Second, is the effect of the image to uplift the viewer? Finally, when brought together do these images provide a diverse perspective regarding kindness, allowing the viewer to transcend their own experiences to see how universal kindness is?

In video format, the Ken Burns effect is applied to these images, which uses a slow pan or zoom to slowly shift the perspective of the image, emphasizing key elements of the image, which is helpful to maximizing attention of the viewer (Wolfe and Horowitz, 2017). Relevant text is variably included to describe the image (using motion graphics). The videos using still images are then blended with other original, kindness-related media, including suggestions, humor, and quotes. A sample of kindness media can be seen here: https://vimeo.com/392331523/b6dfa72edb. Other examples of content can be provided on request.

The reel used in this study was 8-min long and included a mix of individual short videos of various types. All participants were informed that they would receive a $5 gift card for completion of the survey.

### Study Execution

After consenting to participate, each viewer was assigned an identification number and provided a link to an online survey that they would access on their own smart devices. All surveys were anonymous and captured only demographic data (gender, age, race, patient, or staff) and no other personally identifying information.

Baseline self-assessments were solicited for general well-being and for positive and negative affect. Items were taken from a variety of literature exploring meaningful media experiences, using terms frequently employed by investigators examining self-transcendent responses to media (Oliver and Bartsch, 2010; Oliver et al., 2015; Schindler et al., 2017; Rieger and Klimmt, 2019; Zickfeld et al., 2019). Positive emotions included: happiness, feeling calm, grateful, optimistic, and compassionate. Negative emotions included: feeling sad, anxious, and irritated. Scores were on a 1–5 Likert scale with 1 defined as “not at all” and 5 defined as “a lot.” Before baseline questions, all participants were asked: “Overall, how do you feel?”

Following completion of the baseline portion of the survey, participants were provided with instructions through the survey form to watch either the 8-min reel of kindness content OR the standard commercial children’s programming that is pre-selected by staff. The time of watching was recorded on the survey. One study team member was present throughout.

After the 8-min viewing period, study participants completed the same set of emotion survey questions while in the waiting room. In the post-viewing period, participants also self-rated how “moved,” “touched,” or “inspired” they felt after watching the content (on the same 1–5 Likert scale). For those who watched the kindness content, they were asked if they wanted the clinic to continue showing kindness content.

With completion of the survey, all participants were provided instructions on collecting their $5 gift card. Within those instructions, participants were told that they could keep the $5 gift card or donate it to a needy family that attends the clinic. The number of participants who either kept or donated the gift card was captured for each viewing group.

### Data Analysis

Summaries of primary data are expressed as arithmetic mean ± standard error of the mean. Data are displayed as mean (SE) or as frequency. Sub-analyses were undertaken for the parents and staff subgroups.

To examine how changes in emotion from baseline to post-media exposure differed between Kindness Media and Standard television, a mixed ANOVA was employed, with time (changes from baseline) treated as a within-subjects variable, and media condition treated as a between-subjects condition. For within media group comparisons, *post-hoc* tests were conducted using Bonferroni’s adjustment for multiple comparisons.

All positive (happy, calm, optimistic, grateful, and compassionate) and negative (sad, angry, and irritated) emotions were combined and averaged for each subject with reliabilities calculated. The integrated assessments were then analyzed with a mixed ANOVA as described for each individual emotion and *post-hoc* testing with Bonferroni correction.

One-time measures such as moved, inspired or touched were contrasted between each media viewing group using an independent samples *t*-test. Donations and claims of the gift cards were tabulated for each media viewing group and analyzed using Fisher’s exact test.

## Results

A total of 53 parents and staff participated; 50 of them completed the entire protocol. The three who did not complete the study were all parents who had to leave the clinic and were unable to finish.

The 50 participants were the analysis group and their characteristics are summarized in Table 1. Of the 50, 28 were parents and 22 were staff. The majority of participants were female and white. The distributions across Standard and Kindness groups were similar.

**Table 1:** Summary of participant demographics by media type.

Media type	Age (years) [mean (SE)]	Gender distribution	Race	Parents/staff (n/n)
Kindness (*n* = 26)	43 (2)	22F/4M	1A/1B/2H/20 W/2MR	15/11
Standard (*n* = 24)	45 (2)	21F/3M	22 W/2H	13/11

SE, standard error of the mean; F, female; M, male; A, Asian; B, black; H, Hispanic; W, white; MR, multi-racial.

Standard viewers mean self-assessment scores of how they felt were 4.17(0.18) and Kindness viewers rated themselves 4.04(0.17; 1 = “bad”; 3 = “ok”; and 5 = “very good”). These scores indicated that participants, overall, were feeling well and that there were no significant differences between the two media groups, *t*(48) = 0.52, *p* = 0.61.

Tables 2 and 3 summarize the baseline and post-media exposure self-assessments for positive and negative emotions, respectively. As can be seen in Table 2, participants at baseline in both viewing groups started the study in fairly positive states, feeling fairly happy, calm, optimistic, and grateful. These did not differ statistically from one another. However, for the self-report of compassion, those in the Standard viewing group had a lower baseline value than the Kindness viewing group. Most of the people in the kindness media group had indicated baseline self-assessments of 5, the maximal possible score.

**Table 2:** Summary of shifts in positive emotion self-assessments by viewing group.

Affect	Media type	Before	After	Change	Cohen’s d	Interaction *F*	*η*_p_^2^
Happiness						4.77^*^	0.09
	Kindness	4.08 (0.16)	4.50 (0.17)	0.42^*^	0.52		
	Standard	3.63 (0.17)	3.54 (0.18)	−0.08	−0.11		
Calm						17.84^***^	0.27
	Kindness	3.89 (0.18)	4.35 (0.20)	0.46^**^	0.67		
	Standard	4.00 (0.19)	3.54 (0.21)	−0.46^**^	−0.55		
Grateful						8.64^*^	0.15
	Kindness	4.12 (0.21)	4.62 (0.21)	0.50^**^	0.71		
	Standard	3.88 (0.22)	3.63 (0.22)	−0.25	−0.23		
Optimistic						1.55	0.03
	Kindness	3.72 (0.22)	4.16 (0.24)	0.44	0.62		
	Standard	3.33 (0.22)	3.38 (0.17)	0.04	0.03		
Compassionate						0.00	0.00
	Kindness	4.50 (0.21)	4.58 (0.20)	0.08	0.11		
	Standard	3.75 (0.22)	3.75 (0.21)	0.00	0.00		

Before and after reflect pre vs. post media exposure of mean values and corresponding standard error of the mean [Mean(SEM)]. Cohen’s d for repeated measures was used to estimate effect sizes within each row. Results from mixed ANOVA are shown (Interaction *F*), with time (changes from baseline) treated as a within-subjects variable, and media condition treated as a between-subjects condition. The *p* value indicates statistical significance of comparisons for either changes between the Kindness and Standard TV conditions (the Interaction F) or within a viewing cohort (Bonferroni correction).

* *p* < 0.05;

** *p* < 0.01;

*** *p* < 0.001. For full ANOVA results, please see Supplementary Table 1.

**Table 3:** Summary of shifts in negative affect self-assessments by viewing group.

Affect	Media type	Before	After	Change	Cohen’s d	Interaction *F*	*η*_p_^2^
Sad						0.00	0.00
	Kindness	1.39 (0.18)	1.23 (0.17)	−0.15	−0.22		
	Standard	1.83 (0.19)	1.67 (0.17)	−0.17	−0.23		
Anxious						0.78	0.00
	Kindness	1.81 (0.22)	1.54 (0.18)	−0.27	−0.45		
	Standard	1.88 (0.23)	1.67 (0.19)	−0.21	−0.22		
Irritated						1.99	0.04
	Kindness	1.73 (0.22)	1.31 (0.19)	−0.42^*^	−0.43		
	Standard	1.68 (0.20)	1.64 (0.18)	−0.04	−0.11		

Before and after reflect pre vs. post media exposure of mean values and corresponding standard error of the mean [Mean(SEM)]. Cohen’s d for repeated measures was used to estimate effect sizes within each row. Results from mixed ANOVA are shown (Interaction *F*), with time (changes from baseline) treated as a within-subjects variable, and media condition treated as a between-subjects condition. The p value indicates statistical significance of comparisons for either changes between the Kindness and Standard TV conditions (the Interaction F) or within a viewing cohort (Bonferroni correction).

* *p* < 0.05. For full ANOVA results, please see Supplementary Table 1.

As shown in Table 2, viewers in the Kindness media group had significantly different responses from the Standard media viewers in feeling happy, calm, and grateful (by ANOVA). Within group comparisons showed that viewing Kindness media significantly increased participants’ self-reports of feeling happy, calm, and grateful (*post-hoc* testing with Bonferroni correction, Table 2). Feeling optimistic trended higher after viewing Kindness media (*p* = 0.056, with Bonferroni correction), although this shift was not statistically separable from the Standard viewer cohort. No change was reported in either group for compassion. Full ANOVA results can be found in Supplementary Table 1.

Standard TV viewers had little change in positive feelings with the exception of feeling calm. In Standard TV viewers, there was a noted, significant decrease in feeling calm (*p* < 0.001, with Bonferroni correction).

The aggregate mean changes in self-reported positive feelings were also calculated. The mean of the positive emotions (feeling happy, calm, grateful, optimistic, and compassionate) in the Standard TV viewers was unchanged in response to the 8 min of Children’s television [3.72 (0.15) to 3.57 (0.18)]. In contrast, the mean positive scores for Kindness media viewers increased from 4.07 (0.15) to 4.45 (0.17). Mixed ANOVA result comparing the responses to the two media groups showed significant differences [*F*(1,48) = 9.01, *p* < 0.005, *η*_p_^2^ = 0.16]. The increase in mean positive (combined) emotions within the Kindness Media group (with Bonferroni correction) was significant (*p* < 0.005, *d* = 0.90).

Table 3 similarly displays self-reported negative feelings by each viewing group for the entire cohort. As shown in the table, baseline values for feeling sad, anxious, or irritated were low and comparable in both cohorts. In the Kindness media group, after the 8-min exposure, viewers reported less irritation (*p* < 0.05) and a decrease in self-assessed anxiety that approached statistical significance (*p* = 0.08, with Bonferroni correction). No significant changes occurred in the Standard viewer group. Full ANOVA results can be found in Supplementary Table 1.

The mean negative scores started very low and decreased in both groups (change in Standard: −0.08 (0.13) and Kindness −0.28(0.12)). Within the Kindness media viewing group, this decrease was significant (*p* < 0.01, *d* = −0.58) but not within the Standard media viewers. The changes in aggregate negative feelings across the two groups were not statistically separable from one another (*p* = 0.09; Supplementary Table 1).

### Inspiration, Moved, and Touched

All participants were asked in separate questions how much they were moved, touched, or inspired by what they saw. As “moved,” “touched,” or “inspired” are interrelated terms, a reliability analysis was performed prior to combining them into a single scale, with a Cronbach’s alpha of 0.96. The scores from these three were then averaged for each participant by viewing group into a single inspiration score.

**Figure 1:**
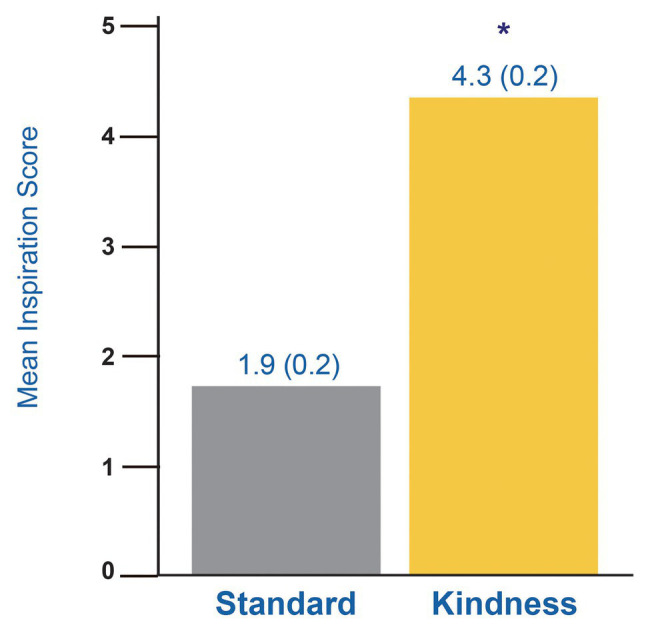
Effect of Standard vs. Kindness Media on being inspired. Each bar reflects viewing group mean of self-reported responses to “feeling touched, moved, or inspired.” ^*^ *p* < 0.001.

Cross media group comparison of these one-time measures were done by independent sample *t*-test. Viewers who watched Kindness media reported significantly higher inspiration scores than those who watched Standard TV [Figure 1; *t*(48) = 8.36, *p* < 0.001, *d* = 2.37].

### Generosity

After completion of the survey, all participants were instructed how to claim their gift cards in the office at that time or informed that they could choose to donate their gift card to a needy family. Table 4 summarizes the choices of participants in each viewing group. Approximately half (54%) of viewers of Standard TV donated their cards, whereas more participants in the Kindness media group (85%) donated theirs. This difference was significant by Fisher’s exact test (*p* = 0.03).

**Table 4:** Participant decision to keep or donate honorarium gift card by viewing group.

Program	Keep	Donate	Total	% Donating	Fisher’s exact test
Kindness	4	22	26	85	*p* = 0.03
Standard	11	13	24	54	
Total	15	35	50		

### Parents and Staff Responses

Table 5 summarizes mean positive and negative emotion scores for parents and staff. As can be seen, the patterns of responses in these subgroups were similar, with some differences noted, including a decrease in positive emotions for parents watching children’s media content that approached statistical significance. Comparing the responses of the two subgroups showed no significant differences.

**Table 5:** Positive and negative affect responses in parents and staff.

	Program	Before	After	Change	Cohen’s d	Interaction *F*	*η*_p_^2^
Positive emotions							
Parents						6.85^*^	0.22
	Kindness	4.07 (0.17)	4.44 (0.21)	0.37	0.97		
	Standard	3.89 (0.19)	3.57 (0.17)	−0.32	−0.37		
Staff						2.25	0.10
		Kindness	4.00 (0.28)	4.40 (0.31)	0.40^*^	0.93		
		Standard	3.51 (0.28)	3.56 (0.31)	0.05	0.09		
Negative emotions							
Parents						0.24	0.00
	Kindness	1.51 (0.19)	1.33 (0.16)	−0.18	−0.74		
	Standard	1.74 (0.19)	1.59 (0.18)	−0.15	−0.53		
Staff						6.81^*^	0.01
		Kindness	1.82 (0.28)	1.39 (0.28)	−0.48^*^	−0.90		
		Standard	1.70 (0.31)	1.74 (0.31)	−0.26	−0.28		

Before and after reflect pre vs. post media exposure of mean values and corresponding standard error of the mean [Mean(SEM)]. These are separated by participant subgroup. Results from mixed ANOVA are shown, with time (changes from baseline) treated as a within-subjects variable, and media condition treated as a between-subjects condition. The significance of change scores from before to after viewing for each program type was tested using least-significant differences tests (Bonferroni correction). Cohen’s d for repeated measures was used to effect sizes within each row. For full ANOVA results, please see Supplementary Table 2. The value of *p* indicates statistical significance of comparisons for either changes between the Kindness and Standard TV conditions (the interaction F) or within a viewing cohort (Bonferroni).

* *p* < 0.05.

The responses to feeling moved, inspired, or touched were also similar across parents and staff. For parents, the mean score after the kindness media was 4.27 (0.19) and for staff was 4.33 (0.30). Following the children’s media, this score was 1.56 (0.23) for parents and 2.39 (0.39) for staff. These differences were different for both subgroups (*p* < 0.001, *d* = 2.36).

Crosstabulations were conducted for parents and staff separately to examine donation behaviors. These analyses showed that a larger percentage of parents (80.0%) and staff (90.9%) donated after viewing the Kindness programming than after viewing Standard television (parents: 53.8%; staff: 54.5%). Because of the limited sample size within the parents and staff subgroups, these differences were not significant. However, they mirror the pattern of results reported for the entire sample, suggesting that the Kindness program was successful in increasing generosity among both parents and staff.

### Qualitative Assessment of Kindness Media

Viewers of the kindness media were also asked if they would like the clinic to continue to show this media. Of the 26 kindness media viewers, 82% responded that they would want the clinic to continue to show it.

## Discussion

This study showed that it is possible to quickly and positively affect the emotional and behavioral states of people in a real-world, healthcare setting. In comparison to the effects of children’s programming, people who viewed the kindness media were happier, calmer, more grateful, and less irritated. They were much more inspired and more generous.

It is noteworthy that there were no changes observed for compassion or for sadness due to the kindness media. Part of the difficulty for each of these variables is that baseline scores were at or very close to the maximum or minimum values, respectively. Overall, at baseline both groups were fairly happy with low levels of self-reported negative emotions in line with previous research on the positivity offset (Ito and Cacioppo, 2005). Despite that baseline state, people responded with further increases in positive emotions as well as small decreases in negative emotions, as reflected by the mean scores for all of the positive and negative emotions.

The results of the present field study are consistent with multiple laboratory-based interventions, namely that simply seeing kindness for a few minutes can elevate viewers, augment happiness (Janicke and Oliver, 2017; Oliver et al., 2015, 2018), as well as affect viewers’ generosity (Schnall et al., 2010). Seeing acts of kindness in the field (as they are occurring, not in media) has also been shown to induce happiness (Rowland and Curry, 2019). The effect of kindness media is also consistent with other studies using reflection or meditation on kindness or acts of kindness to induce happiness (Otake et al., 2006; Fredrickson et al., 2008; Aknin et al., 2015; Zeng et al., 2015; Curry et al., 2018) and connection (Hutcherson et al., 2008), an effect that appears to be cross-cultural (Aknin et al., 2013).

The present study builds on these earlier investigations in several ways. First, the intervention and testing took place in a busy healthcare setting while other potentially distracting events (parents registering children, waiting to be called, watching others being called, etc.) and time pressures were also occurring.

Second, in prior studies investigators generally used publicly available commercial films or moving clips from commercial television, such as *The Oprah Winfrey* show (Schnall et al., 2010). The kindness media that was tested in the present study is an original blend of different types of approaches to express kindness, compassion, and connection. These included still images from around the world converted into video (either a single image or montage of images), motion graphics with suggestions of kindness, humorous depictions of kindness, and concepts in kindness and compassion. While distinct from children’s commercial television, this approach also provides a variety of content that can appeal to a broad range of viewers beyond a single video.

Third, the demographics of participants were also different from prior studies. Most of the previously published work recruited college students as participants. In the present study, middle aged adults (mostly women) were the principal participants, showing that the responses observed in college students are also elicitable in an older group of participants.

Why would kindness media work so well, affecting emotions and behavior within minutes of exposure? Likely because it rapidly taps into or primes implicit (and perhaps explicit) memory for kindness similarly to how media that contains violence, food, sexuality, etc., affects emotions and behavior. This idea, that we are wired to respond to seeing kindness, is also supported by neuroimaging studies that have pointed to specific regions involved in compassion, kindness, and empathy (Eisenberger, 2013; Marsh, 2016; Park et al., 2017). Finally, in concert with the burgeoning literature on endocrine and autonomic changes in response to prosocial behavior (Zak et al., 2007; Goetz et al., 2010; Kok and Fredrickson, 2010; Holt-Lunstad et al., 2019; Kim et al., 2020), collectively this biology and psychology may help explain why kindness is contagious (Keltner et al., 2014).

In a busy world where people are overloaded with “information” input, the ability to elevate people quickly and simply is critical. As the brain processes images very quickly, this neuropsychology can be rapidly tapped into, which, in turn, encourages greater manifestation of the eudaimonic, self-transcendent state that promotes connection (Algoe and Haidt, 2009; Janicke and Oliver, 2017; Oliver et al., 2015). These outcomes, in turn, can help people buffer the response to new stressors. The promotion of connection through elevation also includes how people from different racial groups may view one another (Freeman et al., 2009; Oliver et al., 2015). This has much relevance today, including for health inequity.

Both patients and providers are shouldering a lot of stress. For patients, their stressors can be myriad and interrelated, including concerns over their own health or that of family or friends; how to meet the financial burden of paying for healthcare (APA, 2019), as well as other personal (family, work, etc.) and distant issues (gun control and climate change). Many, particularly those in minority groups, will face discrimination, a potent stressor tied to multiple diseases (Farmer et al., 2019).

Kindness and compassion are major motivators for people to enter the healthcare profession – that is, they want to help people (Mimura et al., 2009). On top of their own personal stressors, however, these professionals must manage a special type of workplace stress that includes solving complex social and medical problems within a short visit as well as documenting the encounter into the electronic health record (EHR). It is no surprise, therefore, that the burnout rate among providers, including both nurses and physicians, is twice that of the general public (National_Academy_of_Medicine_Action_Collaborative_on_Clinician_Well-Being, n.d.). Dentists and dental staff, like physicians and nurses, similarly suffer from a lot of stress and burnout. In 2015, approximately 80% of dentists reported experiencing moderate to severe stress in their practices (American_Dental_Association, 2015).

Although the scientific, technical, and transactional aspects of medicine and healthcare dominate its practice, both ancient wisdom and modern science have shown that compassion plays a key role in healing the sick. As compiled by Trzeciak and Mazzarelli (2019) there are multiple reasons why compassion as expressed by the provider and perceived by the patient is critical to outcomes. From a high-level view, perceived compassion (caring) creates connection and trust. That trust lowers stress and affects patient engagement in their own care, including adherence to a regimen (Greene and Hibbard, 2012; Greene et al., 2015).

There are multiple examples of the impact of burned-out providers and those who are compassionate. Burnout is associated with a greater number of medical errors (Shanafelt et al., 2010). Compassionate providers of care, on the other hand, elicit higher compliance with care, such as cancer screening, control of blood glucose in patients with diabetes, or inflammation and outcomes in patients with Crohn’s disease (O’Malley et al., 2002, 2004, 2012; Hojat et al., 2011; Xu et al., 2020). Finally, compassion interventions, *per se*, may more directly affect disease, such as depression, anxiety, and pain (Mongrain et al., 2018; Austin et al., 2020). Finding ways to instill compassion and elevation in a healthcare setting could, therefore, have meaningful impact.

This study is limited in several ways, in that it was undertaken in a pediatric dental office in a middle-class area with mostly white, female participants who were fairly happy at baseline. Although not directly patients, the parent subgroup is similar to patients in that they are managing the forms that patients would and are likely experiencing concerns or anxiety regarding the outcome of the visit for their children as patients may for themselves. Defining these responses in others who are of different demographic groups as well as patient and provider types is important to understand the potential to affect different subgroups. Although the sample size for parent and provider subgroups was small, the overall indications of efficacy suggest findings would be replicable in a larger setting. In addition, this is an acute response in a modest size study, and while encouraging, needs longer-term studies to gauge its impact, including on health-related outcomes.

Another potential limitation arises from using commercial children’s television in a real-world setting. That is, control participants did not likely view exactly the same set of content due to programming variation. In addition, the format of the kindness media differed somewhat from the children’s commercial television, i.e., many images vs. plot-based stories. In the interest of making a real-world comparison, however, we accepted these differences, including the possibility that some stories about kindness and empathy were shown in the control group. Thus, larger differences may in fact have been observed had we deliberately chosen a more neutral comparison group and excluded pieces that would have been more uplifting.

It is important to note that medical offices for adult patients often play neutral or negative media, including home and garden programs and/or mainstream news. As mainstream news is well-known to rapidly induce stress and anxiety (Johnston and Davey, 1997), medical offices (especially emergency rooms) should carefully consider what is being shown in the waiting rooms and work areas. Beyond television to simply distract the viewer, it could be more beneficial for practices to show inspirational media that can help facilitate positive emotions and interactions for patients and staff.

Altogether, given the agreement with other studies, the potential value of the intervention is significant. Its potential is heightened further as it is low cost, does not require time for training, and can complement other interventions to promote kindness and compassion and positive clinical outcomes. By elevating both providers and patients, we hypothesize that the quality of the interaction will rise, characterized by better communication (e.g., less interruption by provider, more frank disclosure by the patient). That, in turn, can create greater satisfaction of the visit for both. Used over the long-term, it is anticipated that these shifts in the healthcare environment will lead to improvement in outcomes.

## Data Availability Statement

The raw data supporting the conclusions of this article will be made available by the authors, without undue reservation.

## Ethics Statement

The studies involving human participants were reviewed and approved by Integreview IRB (Independent IRB). Written informed consent for participation was not required for this study in accordance with the national legislation and the institutional requirements.

## Author Contributions

DF contributed to the design, conducted data analysis, and wrote the first draft of the manuscript. SU contributed to the design, conduct, and analysis of the study. JM contributed to the design and data analysis of the study. FC contributed to the design of the study and performed statistical analyses. MO contributed to the design of the study and performed statistical analyses. All authors contributed to manuscript revision, read, and approved the submitted version.

### Conflict of Interest

The kindness media was provided by Envision Kindness, a registered not-for-profit with a mission to reduce stress and promote kindness, compassion, joy, and love through kindness media. DF is the President and co-founder of Envision Kindness. He presently serves in that capacity as a volunteer and, as Envision Kindness is a non-profit, has no ownership stake.

The remaining authors declare that the research was conducted in the absence of any commercial or financial relationships that could be construed as a potential conflict of interest.
