# Supplementary material for: Kindness Media Rapidly Inspires Viewers and Increases Happiness, Calm, Gratitude, and Generosity in a Healthcare Setting
Source: Front Psychol. 2021 Jan 20;11:591942. doi: 10.3389/fpsyg.2020.591942 (PMC7854918; doi:10.3389/fpsyg.2020.591942)
Supplement: Supplementary file 1 [file Table_1.docx]

**Kindness Media Rapidly Inspires Viewers**

**and Increases Happiness, Calm, Gratitude, and Generosity in a Health Care Setting**

Article In

*Frontiers in Psychology*

Authors:

David A Fryburg^1^, Steven Ureles^2^, Jessica G Myrick^3^, Francesca R Dillman Carpentier^4^, and Mary Beth Oliver^3^

^1^ Envision Kindness, East Lyme, CT United States

^2^ Children’s Dental Associates of New London County, CT, United States, and School of Dental Medicine, Harvard University, Boston, MA, United States

^3^ Bellisario College of Communications, Pennsylvania State University, University Park, PA, United States

^4^ Hussman School of Journalism and Media, University of North Carolina, Chapel Hill, NC, United States

Correspondence: David Fryburg, MD

Email: [david@envisionkindness.org](mailto:david@envisionkindness.org)

**Supplementary Tables**

Suppl. 1 - Report of media type (Kindness vs. Standard) X Before-After viewing measurement ANOVAs for affective responses

Suppl. 2 - Report of media type (Kindness vs. Standard) X Before-After viewing measurement ANOVAs for overall emotion within parents, staff

**Suppl. 1.** Report of media type (Kindness vs. Standard) X Before-After viewing measurement ANOVAs for affective responses

| Affective Responses | Media Type | Before  Mean (Std. Error) | After  Mean (Std. Error) | After-Before Mean Change | After-Before Cohen’s d | *F* | η_p_^2^ |
| --- | --- | --- | --- | --- | --- | --- | --- |
| Happiness |  |  |  |  |  | Main Effects |  |
| (n = 26) | Kindness | 4.08 (0.16) | 4.50 (0.17) | 0.42* | 0.52 | Before-After = 2.15 | 0.04 |
| (n = 24) | Standard | 3.63 (0.17) | 3.54 (0.18) | -0.08 | -0.11 | Media Type = 11.06** | 0.19 |
|  |  |  |  |  |  | Interaction = 4.77* | 0.09 |
| Calm |  |  |  |  |  | Main Effects |  |
| (n = 26) | Kindness | 3.89 (0.18) | 4.35 (0.20) | 0.46** | 0.67 | Before-After = < 0.01 | < 0.01 |
| (n = 24) | Standard | 4.00 (0.19) | 3.54 (0.21) | -0.46** | -0.55 | Media Type = 1.86 | 0.04 |
|  |  |  |  |  |  | Interaction = 17.84*** | 0.27 |
| Grateful |  |  |  |  |  | Main Effects |  |
| (n = 26) | Kindness | 4.12 (0.21) | 4.62 (0.21) | 0.50** | 0.71 | Before-After = 0.96 | 0.02 |
| (n = 24) | Standard | 3.88 (0.22) | 3.63 (0.22) | -0.25 | -0.23 | Media Type = 5.18* | 0.10 |
|  |  |  |  |  |  | Interaction = 8.64** | 0.15 |
| Optimistic |  |  |  |  |  | Main Effects |  |
| (n = 25) | Kindness | 3.72 (0.22) | 4.16 (0.24) | 0.44 | 0.62 | Before-After = 2.26 | 0.05 |
| (n = 24) | Standard | 3.33 (0.22) | 3.37 (0.17) | 0.04 | 0.03 | Media Type = 4.12* | 0.08 |
|  |  |  |  |  |  | Interaction = 1.55 | 0.03 |
| Compassionate |  |  |  |  |  | Main Effects |  |
| (n = 26) | Kindness | 4.50 (0.21) | 4.58 (0.20) | 0.08 | 0.11 | Before-After = 0.09 | < 0.01 |
| (n = 24) | Standard | 3.75 (0.22) | 3.75 (0.21) | 0.00 | 0.00 | Media Type = 8.47** | 0.15 |
|  |  |  |  |  |  | Interaction = 0.09 | < 0.01 |
| Positive Overall |  |  |  |  |  | Main Effects |  |
| (n = 25) | Kindness | 4.04 (0.15) | 4.42 (0.17) | 0.38** | 0.90 | Before-After = 1.70 | 0.04 |
| (n = 24) | Standard | 3.72 (0.15) | 3.57 (0.18) | 0.15 | 0.19 | Media Type = 7.65** | 0.14 |
|  |  |  |  |  |  | Interaction = 8.84** | 0.16 |
|  |  |  |  |  |  |  |  |
| Sad |  |  |  |  |  | Main Effects |  |
| (n = 26) | Kindness | 1.38 (0.18) | 1.23 (0.17) | -0.15 | -0.22 | Before-After = 2.71 | 0.05 |
| (n = 24) | Standard | 1.83 (0.19) | 1.67 (0.17) | -0.17 | -0.23 | Media Type = 3.78 | 0.07 |
|  |  |  |  |  |  | Interaction = < 0.01 | < 0.01 |
| Anxious |  |  |  |  |  | Main Effects |  |
| (n = 26) | Kindness | 1.81 (0.22) | 1.54 (0.18) | -0.27 | -0.45 | Before-After = 4.70* | 0.09 |
| (n = 24) | Standard | 1.87 (0.23) | 1.67 (0.19) | -0.21 | -0.22 | Media Type = 0.14 | < 0.01 |
|  |  |  |  |  |  | Interaction = 0.08 | < 0.01 |
| Irritated |  |  |  |  |  | Main Effects |  |
| (n = 26) | Kindness | 1.73 (0.22) | 1.31 (0.19) | -0.42* | -0.43 | Before-After = 3.06 | 0.06 |
| (n = 22) | Standard | 1.68 (0.20) | 1.64 (0.18) | -0.04 | -0.11 | Media Type = 0.32 | < 0.01 |
|  |  |  |  |  |  | Interaction = 1.99 | 0.04 |
| Negative Overall |  |  |  |  |  | Main Effects |  |
| (n = 26) | Kindness | 1.64 (0.15) | 1.36 (0.15) | -0.28** | -.58 | Before-After = 9.12** | 0.17 |
| (n = 22) | Standard | 1.73 (0.17) | 1.65 (0.16) | -0.08 | -.20 | Media Type = 0.79 | 0.02 |
|  |  |  |  |  |  | Interaction = 3.03 | 0.06 |

*Note*. Values are from mixed-measures analyses of variance (ANOVA). Before-after measurement is within-subject and media type is between-subject. Before-after refers to assessments measured before and after viewing. Significance of before-after changes within media type assessed with Bonferroni pairwise comparisons. Cohen’s d for repeated measures used to calculate effect size of before-after changes within media type.

^*^*p* < .05; ^**^*p* < .01; ^***^ *p* < .001.

**Suppl. 2.** Report of media type (Kindness vs. Standard) X Before-After viewing measurement ANOVAs for overall emotion within parents, staff

| Overall Emotion | Media Type | Before  Mean (Std. Error) | After  Mean (Std. Error) | After-Before Mean Change | After-Before Cohen’s d | *F* | η_p_^2^ |
| --- | --- | --- | --- | --- | --- | --- | --- |
| Positive Overall |  |  |  |  |  |  |  |
| Parents |  |  |  |  |  | Main Effects |  |
| (n = 14) | Kindness | 4.07 (0.17) | 4.44 (0.21) | 0.37 | 0.84 | Before-After = 0.03 | < 0.01 |
| (n = 13) | Standard | 3.89 (0.17) | 3.57 (0.21) | -0.32 | -0.37 | Media Type = 5.40* | 0.18 |
|  |  |  |  |  |  | Interaction = 6.85* | 0.22 |
| Staff |  |  |  |  |  | Main Effects |  |
| (n = 11) | Kindness | 4.00 (0.28) | 4.40 (0.31) | 0.40* | 0.93 | Before-After = 3.89 | 0.16 |
| (n = 11) | Standard | 3.51 (0.28) | 3.56 (0.31) | 0.05 | 0.09 | Media Type = 2.79 | 0.12 |
|  |  |  |  |  |  | Interaction = 2.25 | 0.10 |
| Negative Overall |  |  |  |  |  |  |  |
| Parents |  |  |  |  |  | Main Effects |  |
| (n = 15) | Kindness | 1.51 (0.19) | 1.33 (0.16) | -0.18 | -0.37 | Before-After = 4.60* | 0.15 |
| (n = 13) | Standard | 1.74 (0.19) | 1.59 (0.16) | -0.15 | -0.53 | Media Type = 1.08 | 0.04 |
|  |  |  |  |  |  | Interaction = 0.02 | <0.01 |
| Staff |  |  |  |  |  | Main Effects |  |
| (n = 11) | Kindness | 1.82 (0.28) | 1.39 (0.28) | -0.42* | -0.90 | Before-After = 4.80* | 0.21 |
| (n = 9) | Standard | 1.70 (0.31) | 1.74 (0.31) | 0.04 | -0.28 | Media Type = 0.08 | < 0.01 |
|  |  |  |  |  |  | Interaction = 6.81* | 0.27 |

*Note*. Values are from mixed-measures analyses of variance (ANOVA). Before-after measurement is within-subject and media type is between-subject. Before-after refers to assessments measured before and after viewing. Significance of before-after changes within media type assessed with Bonferroni pairwise comparisons. Cohen’s d for repeated measures used to calculate effect size of before-after changes within media type.

^*^*p* < .05.
